# Therapeutic uses of oxytocin in stress-related neuropsychiatric disorders

**DOI:** 10.1186/s13578-023-01173-6

**Published:** 2023-11-28

**Authors:** Sen Zhang, Ying-Dan Zhang, Dong-Dong Shi, Zhen Wang

**Affiliations:** 1 Shanghai Mental Health Center, Shanghai Jiao Tong University School of Medicine, 600 Wan Ping Nan Road, Shanghai, 200030 China; 2 Shanghai Key Laboratory of Psychotic Disorders, Shanghai Mental Health Center, Shanghai Jiao Tong University School of Medicine, Shanghai, China; 3 Institute of Psychological and Behavioral Science, Shanghai Jiao Tong University, Shanghai, China; 4 College of Physical Education and Health, East China Normal University, Shanghai, China

**Keywords:** Oxytocin, Stress-related neuropsychiatric disorders, Neural circuits, Depression, Anxiety

## Abstract

Oxytocin (OXT), produced and secreted in the paraventricular nucleus and supraoptic nucleus of magnocellular and parvocellular neurons. The diverse presence and activity of oxytocin suggests a potential for this neuropeptide in the pathogenesis and treatment of stress-related neuropsychiatric disorders (anxiety, depression and post-traumatic stress disorder (PTSD)). For a more comprehensive understanding of the mechanism of OXT's anti-stress action, the signaling cascade of OXT binding to targeting stress were summarized. Then the advance of OXT treatment in depression, anxiety, PTSD and the major projection region of OXT neuron were discussed. Further, the efficacy of endogenous and exogenous OXT in stress responses were highlighted in this review. To augment the level of OXT in stress-related neuropsychiatric disorders, current biological strategies were summarized to shed a light on the treatment of stress-induced psychiatric disorders. We also conclude some of the major puzzles in the therapeutic uses of OXT in stress-related neuropsychiatric disorders. Although some questions remain to be resolved, OXT has an enormous potential therapeutic use as a hormone that regulates stress responses.

## Introduction

“Amor est vitae essentia”, as a proverb states, “love” is consistent with “pleasure” in neuroscience. Oxytocin (OXT), an ancient, conserved, “love” related neuropeptide, is an endogenous hormone consisted of 9 amino acids and is well-known for its effect on lactation and parturition in mammals. It’s now over one century since pharmacologist *Sir* Henrry Hallet Dale discovered that a pituitary extract can promote uterotonic activity and be named by “Oxytocin” meaning “quick birth” [1]. OXT was synthesized by magnocellular and parvocellular neurons in the paraventricular nucleus (PVN) and supraoptic nucleus (SON) of the hypothalamus, and transported by the posterior pituitary into the circulation [2–4]. At whole brain level, the distribution of OXT receptor (OXTR) is mainly in different brain regions, especially in the projection of magnocellular and parvocellular neurons, including hippocampus, amygdala, prefrontal cortex [5–7].

Stress is always a natural response of organism to environmental disturbances, which is often associated with the experience of the emotional and physiological challenge. The chronic stress environment may impair individual mental health and induce depression, anxiety, fatigue, PTSD [8]. The physiological and pathophysiological mechanism of OXT have attracted sustained research interest given its role in complex behavioral traits and in the mental health conditions [3]. In our previous work, we found that OXT may be a modulator of stress-related neuropsychiatric disorders [9].

Here, we reviewed recent advances in preclinical and clinical study regarding the potential use of OXT in stress-related neuropsychiatric disorders. For a more comprehensive understanding of the mechanism of oxytocin's anti-stress action, we summarized the signaling cascade of OXT binding to targeting stress. Then the advance of OXT treatment in depression, anxiety, PTSD and the major projection region of OXT neuron were discussed. The efficacy of endogenous and exogenous OXT in stress responses were highlighted in this review. Finally, current biological strategies to augment the level of OXT in stress-related neuropsychiatric disorders were summarized, shedding a light on the treatment of stress-induced psychiatric disorders.

### Oxytocin receptor coupled signaling cascade

Understanding how OXT mediated a signaling cascade under stress is a biochemical basis for attenuating the neuropsychiatric disorders. OXTR, as a G protein-coupled receptor (GPCR) consisting of 389 amino acids, plays a vital role in maternal behavior, social behavior, and parturition [10]. And OXTR is coupled to the heterotrimeric complex of G proteins, including the G_α_, G_β_, G_γ_ subunits [11]. In general, OXTR mediates Ca^2+^ release and transport pathways. When Gβ and Gγ subunits bind to OXTR, activation of Gα enhance the activation of phospholipase C (PLC) and downstream inositol 3 phosphate (IP3) and 1,2-dicyaglycerol (DAG), inducing Ca^2+^ release from the endoplasmic reticulum [12]. Elevated intracellular Ca^2+^ ions concentration forms a complex with calmodulin protein and induces smooth muscle contraction, which may be involved in myometrial cells contraction during labor [13, 14].

OXT-mediated elevation of intracellular Ca^2+^ concentration was associated with activation of IP3-sensitive Ca^2+^ storages of astrocytes in vitro [15]. OXT also induces Ca^2+^ influx by regulating transient receptor potential vanilloid type 2 (TRPV2) channels to induces anxiolytic activity in male rats [16]. The process of OXT-induced intracellular Ca^2+^ changes may also be involved in the regulation of neuronal excitability or synaptic plasticity, but depends on the type of neuronal cell [16]. In the paraventricular OXT neurons, OXTR mediates increased neuropeptide S synthesis and induces anxiolysis [17]. In addition, it was found that OXTR interacts mitogen-activated protein kinase (MAPK) signaling pathway by acting with epidermal growth factor receptor (EGFR) [18]. And OXTR-EGFR signaling promotes the maintenance of LTP in the CA1 region synapses of the hippocampus of female and male rats [19].

**Fig. 1:**
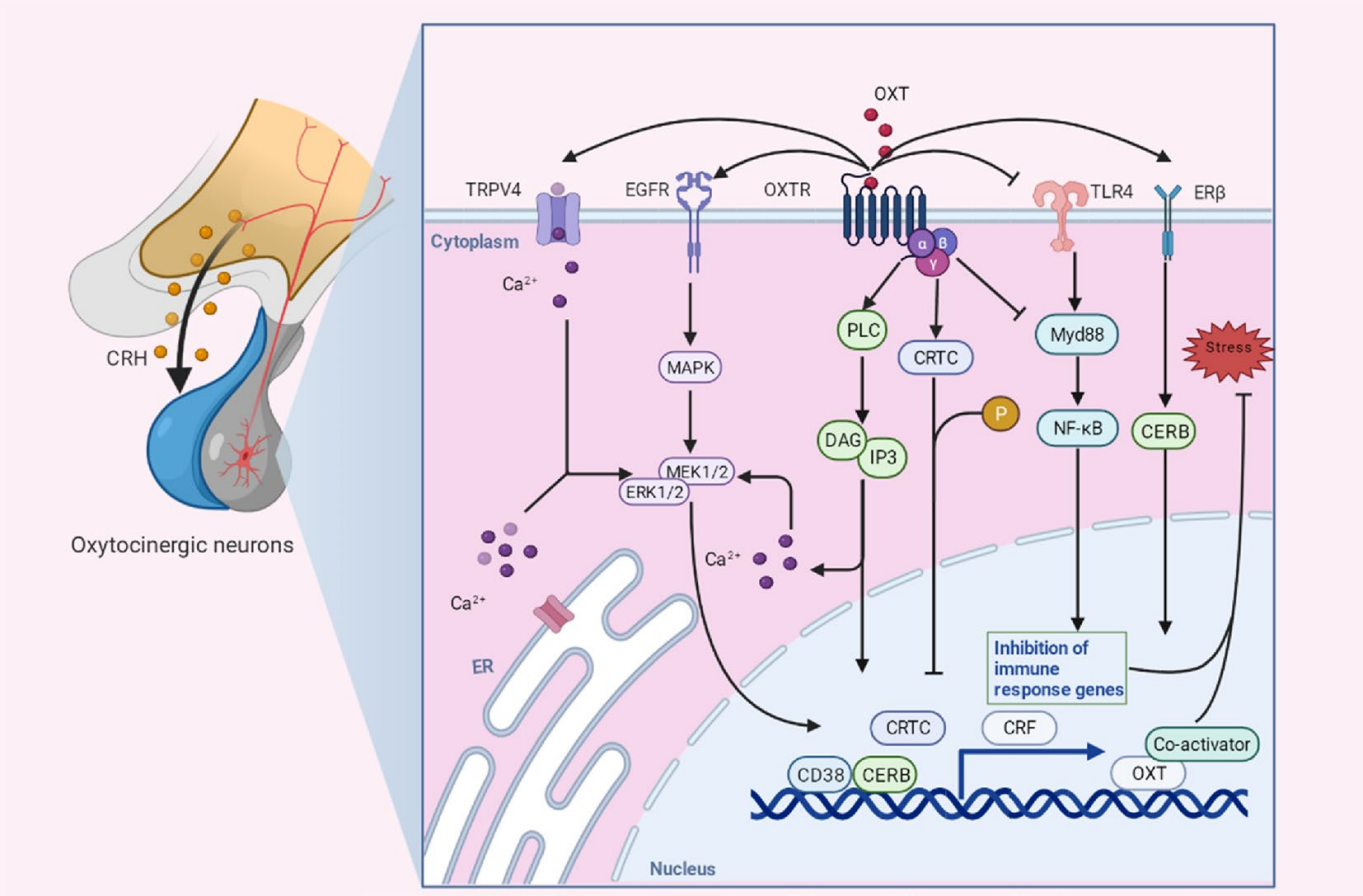
Schematic description of the signaling cascade of Oxytocinergic neurons in PVN. OXT binding to OXTR stimulates the transcription of EGFR, which leads to subsequent MAPK pathway activation. The Ca^2+^, influx into the cytoplasm via activated TRPV4 and release from PLC dependent pathway, interacts the ERK1/2 and MEK1/2 to regulate the subsequent transcription factor (CREB, CTRC, CD38). Furthermore, OXT binding also suppress the TLR4 mediated signaling cascade, which causes the inhibition of immune response genes (TNF-α, IL-1β, NLRP3). When ERβ is activated, intracellular cascades promote the transcription of OXT via CREB and coactivators. These signaling cascades ultimately buffer the molecular changes induced by stress. Image created with Biorender.com

The distribution characteristic of OXTR mediate the physiological effect in the stress response. In hypothalamic corticotrophin-releasing hormone (CRH) neurons, restraint stress induces the CRH expression and cAMP response element-binding (CREB) translocation into nuclear in rat [20]. As a co-activator of CREB phosphorylation, CREB-regulated transcriptional coactivators (CRTC) dephosphorylates and enters the nucleus to interact with the CRF promoter, activating CRF gene expression in the stress [21]. Moreover, Estrogen receptor-mediated signaling also interacts with the OXTR signaling cascade. Immunohistochemistry and in situ hybridization results revealed that estrogen receptor beta (ERβ) was highly expressed in hypothalamic OXT neurons in rats and mice [22, 23]. Interaction between hypothalamic ERβ and OXT can modulate anxiety behavior and HPA axis activity in restraint-stressed rats [24]. And treatment with ERβ ligand agonist, 5α-androstane, 3β,17β-diol (3β-diol), upregulates the expression of OXT mRNA and promotes OXT transcription via CREB and steroid receptor coactivator-1 [25]. In particular, estrogen withdrawal induces hypothalamic oxytocin neuronal plasticity and increases anxiety behaviors via enhancing OXT transmission between the PVN and dorsal raphe nucleus [26]. In addition, androgen receptors have been found to co-localize with hypothalamic OXT by immunocytochemistry in the postmortem brain of patients with mood disorders [27]. The application of techniques for the study of co-expression of OXT and other hormone receptors is still limited and remains at the level of in situ hybridization or immunohistochemistry only. The utilization of single-cell transcriptomics, spatial transcriptomics, and the development of other hormone receptor sensors may provide technical support to address this issue. Spatial transcriptomics (ST) is a technique to understand gene expression differences between tissues, organs and pathological states by describing the gene expression profiles of specific cell types through spatial dimensionality analysis, which is capable of resolving transcripts at different spatial locations in tissues [28]. This could help understand the mechanisms by which signaling pathways activated by oxytocin and other hormone receptors produce different behavioral paradigms in different brain regions. In addition, the application of single-cell transcriptomics has enabled the observation of tissues at single-cell resolution. Computational tools have been developed to estimate cell–cell communication activity from RNA-seq data using signaling databases [29, 30]. A recent study analyzed multiple error robust fluorescence in situ hybridization (MERFISH) data from the mouse hypothalamic preoptic region, featuring 161 genes and 73,655 cells [31]. Self-regulation of excitatory neurons and modulation of inhibitory neurons by excitatory neurons through OXT signaling were found in all slices, a result consistent with the known primary function of OXT signaling [31]. Further analysis identified localized regions of high activity and spatial orientation of OXT signaling, which is consistent with protein staining of OXT and its receptors [31]. A gradual change in the direction of predicted OXT signaling and in the region of high activity was observed through adjacent slices [31]. Notably, whole-brain quantitative expression of OXTR is important for understanding expression specificity in temporal and spatial patterns. Kyra et al. constructed quantitative expression changes of OXTR during postnatal development and at the whole-brain level using OXTR fluorescent reporter mice (OXTRvenus/+) [32]. They also built an open OXTR web-based visualization data (https://kimlab.io/brain-map/OTR/), which provides an anatomical basis for subsequent OXT/OXTR-mediated behavioral and neural circuits differences across age [32]. A clear pattern of OXTR gene expression in the developing brain was found in postmortem human brain samples, with OXTR expression increasing during the prenatal period, peaking in early childhood, and strong spatiotemporal coupling to psychiatric disorders [33]. Brandon et al. revealed spatial and temporal enrichment of OXTR expression in adult neurons of the mouse olfactory bulb, with OXTR expression peaking during activity-dependent integration [34]. Using viral labeling, confocal microscopy, and cell type-specific RNA-seq, they demonstrated that OXTR signaling promotes morphogenesis and synapse maturation by regulating newly integrated adult neurons [34]. Therefore, analyzing OXT and its related receptors from a temporal and spatial perspective may provide a more precise strategy for application. Furthermore, the stress response is often accompanied by overactivation of the neuroimmune system. OXT (i.c.v injection) ameliorated the negative effects of maternal separation (MS) on the hippocampal neuroimmune system and reduced inflammatory cytokine via suppressing the Toll like receptor 4 (TLR4) pathway (IL-1β, Myd88, TNF-α, NLRP3) [35]. Increased OXTR expression is regulated by NF-κB downstream of TLR4 and attenuates LPS-induced macrophage inflammatory response [36]. Collectively, these findings shed light on the role of the interaction of OXTR-mediated signaling cascades with other signals in PVN (Fig. 1).

Given the molecular structure of OXT, it’s usually harder to cross the blood–brain barrier. Therefore, OXT is administered by nasal spray and diffuses in the cerebrospinal fluid through the olfactory bulb axonal projections rather than through the blood–brain barrier [37, 38]. It should be mentioned that the hypothalamus is the first region to respond to peripheral physiological changes, such as starvation and cold. Hypothalamus has permeable microvessels attached and nuclei with looser tight junction structures for transit of hormones (i.e., leptin, ghrelin etc.), such as the medial basal hypothalamus and arcuate nucleus [39, 40]. It has been shown that the median eminence (ME), located in the medial basal hypothalamus, near the arcuate nucleus, is the entry point for some neuropeptide hormones and circulating signals into the brain via the fenestrated capillaries [40, 41]. Leptin is a 16 kDa peptide hormone that maintains energy homeostasis by activating leptin receptors (LepR) in the brain [42]. Previous studies have suggested that leptin is unable to enter the brain through the blood–brain barrier, but Manon et al. found that LepR are expressed in hypothalamic ME tanycytes and respond to leptin [43]. Leptin can be transported by transcytotic route across tanycytes, and the LepR mediates the process of leptin internalized, which is necessary for transport into the cerebrospinal fluid [43]. The above results suggest that leptin can enter the brain through routes outside the blood–brain barrier to participate in the control of feeding and the maintenance of energy homeostasis [43]. It is still unclear whether OXT also enters the brain from fenestrated capillaries of the medial basal hypothalamus similarly to leptin. However, it has been found that the nose-to-blood route is more critical for the pharmacological effects of nasal OXT than the nose-to-brain route, and that nasal OXT produces its effects by increasing peripheral blood concentrations rather than directly entering the brain in the human subject [44]. In addition, Chen et al. also confirmed that oral and nasal spray OXT increased peripheral circulating concentrations [45]. OXT selectively enhances C-touch fiber-targeted pleasurable responses to social touch processing via peripheral pathways rather than directly into the brain [45]. And OXT-deficient secreting mice (*Oxt*^*−/−*^, *Cd38*^*−/−*^, *Oxtr*^*−/−*^) exhibit deficits in maternal bonding behavior, whereas peripheral OXT delivery results in the restoration of maternal bonding behaviour, implying that circulating OXT may cross the blood–brain barrier [46]. Further, OXT is transported to the brain via the receptor for advanced glycosylation end products (RAGE) on brain capillary endothelial cells and validated in RAGE-deficient mice (*Ager*^−/−^) [46, 47]. In addition, the nanoparticle formulation also enables sustained delivery of peripheral OXT to the brain [48]. The application of nano-delivery systems not only improves the infiltration of OT in the brain, but also prolongs the half-life of OXT by encapsulation and sustained release [49]. The above results imply that OXT's role in entering the brain is important for pharmacological intervention in stress-related neuropsychiatric disorders, but more clinical studies are needed to verify this.

### The major projection of intracerebral oxytocin in stress

#### Hippocampus

The hippocampus is usually considered to be a vital brain region that regulates emotion, cognition and particularly vulnerable to stress exposure. And OXTR is abundantly distributed in the hippocampus, suggesting that the OXT system may mediate the hippocampal regulation of stress responses [50]. Using double-labeled confocal immunofluorescence images, Lin et al. found that OXTR is mainly expressed in pyramidal neurons in the CA2/3 and dorsal DG region in OXTR-Venus knock-in mice [51]. The expression and binding of OXTR is detectable in subregions, such as CA1, CA2, CA3 and dentate gyrus (DG) of hippocampus in rodents [12]. In stressed brain, there are three potential mechanisms mediated the regulation of OXTR signaling in hippocampus, including regulation of neuronal excitability, neurogenesis, and regulation of glucocorticoid level.

Whole-cell recordings showed that activation of OXTR led to an increase in the frequency and amplitude of spontaneous inhibitory postsynaptic currents by evoking GABAergic interneurons [52]. And Silvia et al. also suggested that OXT exposure inhibit hippocampal glutamatergic neurons and maintain the balance of physiological excitation/inhibition in vivo and vitro [53]. In hippocampal slices, OXT induced membrane depolarization and enhanced electrically triggered action potential discharges in CA3 pyramidal neurons, indicating neuronal excitability occurred in CA3 pyramidal neurons in hippocampus [54]. Taken together, these studies identified that OXT mediated pathway can maintain the homeostasis of neuronal excitability.

Adult neurogenesis refers to the generation and proliferation of new neurons, while stress often negatively regulates hippocampal neurogenesis [55]. To the best of our knowledge, there is currently no evidence for OXT on hippocampal neurogenesis in the human brain, thus we mainly focus on the mammalian brain to discuss the OXT mediated neurogenesis in hippocampus [56]. Peripheral OXT administration (1 ng/μl) significantly enhanced the BrdU-labeled cells in the ventral DG, indicating OXT injection increased adult-generated cells in neurons and glia when animal exposure to acute cold-water stress [57]. OXT treatment was also accompanied by a phenotype of hippocampal cell proliferation and dendritic maturation [58]. Further research discovered that PVN oxytocinergic signaling promotes neurogenesis in the sub-granular zone of DG by activating OXTR in pyramidal neurons in the CA3 region of the hippocampus [54].

Furthermore, previous studies pointed out that systemic administration of OXT reduces glucocorticoid receptor mRNA expression in hippocampal CA1, CA2 and DG of rat [59]. The high concentrations of stress-induced corticosteroids (CORT) level reduced hippocampal synaptic plasticity by activating glucocorticoid receptors, which may be a hindrance to NMDA receptor-dependent synaptic plasticity [60, 61]. These studies all showed that stress affects the hippocampus and behaviors through the regulation of glucocorticoid.

#### Prefrontal cortex

Prefrontal cortex is a key target region in stress-related neuropsychiatric disorders. In rodent, acute restraint stress triggered the reinstatement of methamphetamine conditioned place preference induced by methamphetamine, whereas microinjection of OXT can reverse it via the glutamatergic system of mPFC [62]. In the rodent model of PTSD, single prolonged stress (SPS) stimulated the expression of neuroinflammation factors (i.e., IL-1β, IFN-γ) in mPFC, hippocampus, and amygdala [63]. But intranasal OXT administration effectively ameliorated SPS-induced fear extinction recovery through anti-inflammatory effects and reduced the higher CRHR1 protein levels in mPFC and amygdala [63, 64]. Using c-fos immunofluorescence labeling and quantification, the hypo-activation of prefrontal cortex and amygdala neurons were found in vulnerable rats with a robust PTSD syndrome including increased anxiety and decreased arousal. These phenomena can be rescued by intracerebral infusion of OXT via emotional remodeling [65]. Nevertheless, there are still some studies revealing the mechanism of OXT's intervention of stress by targeting the prefrontal cortex at different levels. Blood OXTR mRNA is not only thought as a potential biological biomarker in the prefrontal cortex of hyporesponsive HPA axis subtype of PTSD, but also likely regarded translational evidence that the HPA axis response specificity is dependent on the regulation of OXTR expression [66]. Likewise, OXTR-mediated ERK and MAPK phosphorylation in mPFC relieved the neonatal maternal separation-induced social deficits behaviors [67]. Interestingly, in the early paternal deprivation (PD) experiences, optogenetic activation of OXT neurons in PVN projected to the prelimbic cortex can reverse PD-induced emotional and social preference behaviors [68].

Furthermore, OXT plays a specific regulatory role in the prelimbic subregion of mPFC and produces anxiolytic phenotype via interact with GABAergic neurons [69]. Meanwhile, OXT neuron-specific circuits in the cortex also have sex dependence in interventions for anxiety. Using optogenetic techniques and electrophysiological recordings, OXTR interneurons (OXTRINS) of mPFC was found to modulate anxiety behavior in male mice, but not in female mice [70]. This process may be associated with corticotropin releasing hormone binding protein (CRHBP), an OXTRINS-specific expressed protein, blocking CRH-induced activation of pyramidal 2/3 neurons of mPFC. Surprisingly, our previous work also demonstrated that mPFC has been activated in maternal separation (MS) [9]. Among them, unpredictable MS impaired the structural and functional glutamatergic synapses in pyramidal neurons of mPFC and increased stress susceptibility [9]. And RNA-sequencing revealed that the OXT gene played a crucial role in predictable MS-induced stress resilience [9].

The obvious studies have proved the specific mechanism of action of OXT and neurons in prefrontal cortex, but the OXT mediated biological mechanism and the change of subregion of prefrontal cortex in other stress model still need more evidences. In the clinical studies, the effect of intranasal OXT administration on different stress groups is usually related to the functional connectivity between the prefrontal cortex and other brain regions. Early life stress alters amygdala-prefrontal functional connectivity and sensitivity to the effects of OXT treatment [71]. In PTSD patients, OXT may reduce anxiety and fear expression in the amygdala in PTSD by increasing control of the ventromedial prefrontal cortex (vlPFC) over CeM (males) or by reducing salience processing of dorsal anterior cingulate cortex over BLA (females) [72]. In trauma-exposed individuals, amygdala responses to fearful faces were significantly enhanced after a single dose of OXT, and amygdala-ventromedial and vlPFC connections were weakened [73]. Therefore, repeated intranasal injections OXT is a promising early preventive intervention for individuals with increased risk of PTSD due to severe acute symptoms [73]. In addition, the results also showed that PTSD patients with the treatment of OXT have increased connectivity between the dorsolateral prefrontal cortex and anterior cingulate gyrus in working memory and control system connectivity compared to placebo [74]. And OXT use was found to affect the functional connectivity of the BLA and vlPFC hindering the functioning of emotion regulation networks in response to trauma-exposed situation [75]. This suggests that in traumatized individuals, intranasal OXT administration needs to be administered more cautiously to intervene in PTSD.

#### Amygdala

Previous evidence supported that OXT can attenuate stress-induced fear responses by targeting the amygdala. Excessive fear expression may be associated with the development of anxiety disorders such as PTSD, panic disorder, and phobias. It was found that amygdala neurons participate in the emotional remodeling of OXT through morphological changes, but cannot be ruled out the non-specificity in other brain regions, such as prefrontal cortex [65]. Traumatic experiences can lead to abnormal fear extinction due to the biological imbalance between HPA and amygdala fear circuit. Therefore, intranasal OXT administration restores the impaired prosocial behavior of male rats under SPS stimulation and the expression of amygdala OXTR by regulating CRHR sensitivity [64]. Interestingly, the amygdala not only played a role in trauma-related stress, but also mediated the efficacy of OXT in social stress-induced avoidance behavior in prairie female voles [76]. In autistic mice, OXT intranasal administration improved microglia activation in the amygdala and reduced neuroinflammation and oxidative stress [77].

Additionally, centralof the amygdala (CeA) has been shown to be involved in the stress regulation of OXT and OXTR or GABA receptor are enriched in this region [78]. In forced swimming stress, OXTergic neurons of CeA regulated stress-coping behavior, and the mechanism may be through the inhibition of excitatory amino acids [78]. OXT microinjection in CeA promoted social preference and reduced the anxiety levels, whereas CeA infusion with OXTR-antagonist dose-dependently reduced sociality and increased anxiety [79]. But administration of the GABAA receptor antagonist bicuculline in combination with OXT to the CeA blocked the effect of the anxiolytic property of OXT [79], suggesting that OXT interacts the GABAergic neurons of CeA in anxiolytic effect. In the long-term isolation stress, OXT ameliorated depressive and anxious behaviors via increases miniature inhibitory postsynaptic currents in CeA neurons, indicating that OXT rescues glutamatergic synaptic transmission in CeA of isolated mice [80]. In recent study, Francesconi et al. found that OXT also projects to CeA through type II bed nucleus of the stria terminalis (BNST) neurons by recording retrogradely labeled [81]. And they found that OXT increases the frequency of spontaneous inhibitory post-synaptic currents in type II BNST → CeA output neurons, suggesting that OXT inhibits BNST → CeA neurons to promote cued fear stress [81]. Hence, these studies suggested that OXT mediate the neuronal excitability of CeA in the improvement of trauma and fear related stress, but still need more research to elucidate biological mechanism.

### Oxytocin in regulation of stress responsivity

#### Oxytocin regulates the HPA axis in response to stress

The relationship of OXT activation and HPA axis hormone interactions are critical to elucidate the regulation of OXT in stress. Among changes of stress-related hormone levels, the feedback inhibition of corticosteroids is mediated by OXT signaling pathway and related to CRH, which is called the main driver of HPA axis [82, 83]. Intracerebroventricular (i.c.v) injection of CRH may act directly or indirectly upon magnocellular neurons to facilitate OXT release in rat [84]. Then, using double labelling in situ hybridization, Arima et al. observed the localization of CRH receptors type-1(CRHR1) and type-2(CRHR2) in the SON and PVN, suggested the colocalization of CRHR2 with OXT mRNA in the SON [85]. Consistent with the above findings, CRHR1 antagonist significantly attenuated the stress-induced alteration of OXT, CORT and displays anxiolytic effects in rats [86]. Thus, antagonism of CRHR may be necessary for the release of OXT in stress. Interestingly, OXT receptor interneurons are involved in regulate anxiety-related behaviors via specifical expression of CRHBP, an antagonist of CRHR [70]. In a rat model of PTSD, it is in line with the above evidence that the CRHR regulation of anxiety was sensitive to OXT signaling via OXT antagonist atosiban [64]. Among these, the expression of CRHR1 was more sensitive than CRHR2 and the higher protein level was found in the amygdala in SPS, while the OXT signaling pathway demonstrated a therapeutic specificity to the amygdala, indicating that the amygdala OXT signaling pathway may amended SPS induced PTSD by regulating CRHR1 [64]. Therefore, the CRHR plays a critical role in regulation of OXT in the stress response and cause a cascade reaction of HPA axis.

Previously, central OXT administration has been implicated in downregulation of HPA responses to noise stress and inhibition of CRF and CORT release in rodents [87]. There is a study also showed that the i.c.v OXT injection after oophorectomy reduced plasma ACTH concentrations and the expression of CRH mRNA in the rat’s hypothalamus after stress stimulation [88]. Treating with an OXT antagonist, male marmosets expressed significantly higher HPA-axis activity across the stressor compared with saline, indicating that the OXT system reduces the stress-induced cortisol secretion [89]. Hence, the fluctuations of both endogenous and exogenous OXT level are reflected the resilience of stress response and resistance to stress related hormone.

It should be noted, OXT may act as a buffer in stress induced HPA axis response via prosocial behaviors. Previous findings supported those social behaviors act as a buffer for the imbalance between HPA axis and stress responses, and OXT is considered as mediator in the buffering of social [90, 91]. To further confirm mediation effect of OXT, Smith and colleagues first demonstrated that OXT release in PVN and OXT neurons modulate HPA axis responses and alleviate immobilization stress induced anxiety-like behavior in the prairie vole (a socially monogamous rodent for long term pair bonds) [92]. Similarly, consolation behavior, a common empathetic response and a prosocial behavior, can make prairie vole to increase grooming in stress partner, suggesting that they provide a social buffering when partner experience an unobserved stressor [93]. Exposure to the stress, the level of CORT and the ACC activity were increased in the prairie vole [93]. And i.c.v injection of OXT receptor antagonist induced the previous molecular changes and abolish the benefit of consolation behavior [93]. Therefore, OXT can improve the stress induced dysfunction of HPA axis via a social behavior dependent way. But there are growing evidences presented that OXT sometimes cannot attenuate the anxiety behavior and even led to an increase in social anxiety and aggressive behaviors [94, 95]. To explain this phenomenon, the social salience hypothesis of OXT argued that OXT regulates the salience of social cues, like competitive, cooperative or stress environment, which modulates individual’s responses though circuit-specific action [96]. This hypothesis proposes that OXT increases the salience of safety signals in positive situations (e.g., empathy, trust, cooperation etc.), thereby improving stress responses [97]. Conversely, OXT induces a bias toward negative social cues in the unpredictable threatening situations (e.g., competition, scare, aggression) and increases stress responses [95]. These results show that OXT neurons play a critical role in regulating stress responses, especially in the social buffering. The level of OXT showed resilience in regulating stress response, and the difference may be caused by the salience of social cues.

#### The exogenous and endogenous oxytocin efficacy in stress responses

The OXT system was greatly activated by stressful or threatening condition, and either endogenous or synthetic oxytocin produces stress resistance. In general, the discussion of oxytocin mainly focuses on the intervention effect of exogenous synthetic OXT administration on stress state, and the expression level of endogenous OXT in anxiety and stress [12, 98]. There are several preclinical and clinical studies suggested that exogenous administration of recombinant OXT exerts strongly anxiolytic, stress resistant, social-buffering effect on humans and rodents (Table 1). Various animal studies aimed to uncover the role of synthetic OXT in anxiolytic effect and stress response, even though the mechanism of action varies. However, in mice, the alterations in anxiety-behavior were described to be controversial, with elevated stress response in mild stress model, which may dependent on the instability of behavior of rat or the dose of OXT (chronic i.c.v OXT injection) [99]. Similarly, chronic high-dose i.c.v OXT administration (over 14 days) produced an anxiety-like behavioral phenotype and reduced the expression of OXTR levels, whereas chronic low-dose OXT prevented hyper anxiety and reduced ACTH sensitivity and adrenal hypertrophy [100]. These results suggest that the dose-dependent effect of OXT needs to be revisited in the application of stress-induced psychiatric disorders [100].

**Table 1:** The efficacy of exogenous administration of OXT in different stress

Species, gender	Stress type	Dosage Regimen	Index change	Outcome		References
Rat, M	Chronic stress	Oral treatment, 10 IU/400 μl, 14 days	NAT, VMAT2↑ catecholamine↓	Attenuated adrenal gland atrophy		[157]
Rat/mice, M&F	Acute restraint stress	I.c.v injection in PVN, rats1 nmol/2 μl; mice 0.5 nmol/2 μl	OXTR↑ Crf mRNA, CRF, CREB, CRTC3↓	Regulating stress response		[158]
Rat, M	Early-life stress Restraint stress	I.c.v injection in mPFC and PVN, 1 µg/µL	Duration in central zone, open arm ratio ↑	Anxiolytic effect		[9]
Rat, M&F	Mild stress	I.c.v injection, 10 ng/h, duration 14 days	CRFR2α form in CSF ↑	Anxiety-like behavior		[99]
Rat, M	Tail-shock stress	Intranasal administration, 1 mg/ml, 200μL	fEPSP, LTP↑	Synaptic plasticity↑		[159]
Rat, M	PTSD	Intranasal administration, 1 μg/μL, 2 × 10 μL	CRHR1 in mPFC↓	Prosocial contacts↑		[64]
Mice, M	Early life stress	Intranasal administration, 2μL, 12 IU/kg	Serum CORT↓	Paw-licking behaviour↑ Self-grooming↑		[160]
Mice, M	Chronic subordinate colony housing stress	I.c.v injection, 1 ng/h, 10 ng/h	OXTR(high dose) ↓ ACTH sensitivity ↓	Anxiety (high dose) Inhibit hyper-anxiety (low dose)		[100]
Mice, M&F	Acute restraint stress	I.c.v injection, 1.25 or 2.5 μg/0.5 μL	Adult hippocampal neurogenesis↑	METH addiction, stress response↓		[161]
Mice, M&F	Acute & chronic Social defeat	Intranasal administration, 8 IU/kg	Social interaction (F)↓ social interaction (M)↑	Anxiolytic in male		[162]
Mice, M&F	Chronic neuropathic pain	I.c.v injection, 100 μM/0.5μL	Pre-LTP in ACC↓ action potential, resting membrane potential↑	Attenuates neuropathic pain and emotional anxiety		[163]
Prairie voles, F	Chronic social isolation	Subcutaneous injection, 20 μg/50 μl, 14 days	**/**	Sucrose preference↑ Immobility in FST↓		[164]
Prairie voles, F	Chronic social isolation	I.p injection, 0.05 mg/kg, 0.5 mg/ml	Serum CORT↓	Oxidative damage, Telomere degradation↓ Sucrose preference↑		[165]
Prairie voles, F	Mild stress	I.c.v injection in PVN, 10 ng/nl	PVN GABA activity↑ HPA axis activation↓	Anxiolytic effect		[166]
Marmoset, M&F	Chronic social isolation	Intranasal administration, 25 IU	Serum CORT in female↓	Modulate HPA-axis response via prosocial behavior		[89]
Human, M	Acute psychosocial Stress	Intranasal administration, 24 IU	Amygdala–hippocampal functional connectivity ↑	Stress reactivity and sensitivity ↓		[167]
Human, M	Acute psychosocial Stress	Intranasal administration, 24 IU	Limbic deactivations↑	Stress reactivity↑		[168]
Human, F	Experimentally induced pain	Intranasal administration, 24 IU	Heart rate variability↓	The salience of social proximity↑		[169]

It is also worth noting that changes in endogenous OXT levels and neuronal activities during stress, which may lead to the identification of biomarkers for the diagnosis of stress-related neuropsychiatric disorders (Table 2). Generally, the oxytocin system of the hypothalamus is activated both in human and rodent, and even peripheral OXT levels are affected by stress. But the opposite results were found that the OXT system plays a buffering role in reducing the sensitivity of stress responses [101]. The above results indicate that endogenous changes in OXT expression may be safer and more efficient in stress regulation than peripheral OXT administration, but these are based on the normal endocrine system of the oxytocin system.

**Table 2:** The endogenous changes of OXT in stress

Species, gender	Stress type	Outcome	References
Mice, M&F	Chronic variable stress	Basal CORT and PVN immunoreactivity↑ CRH and OXT mRNA in the PVN↑ Without anxiety behavior	[170]
Mice, M&F	Chronic neuropathic pain	OXT concentration in PVN and elevated expression of OXTR in ACC	[163]
Rat, M	Restraint stress	Arterial pressure, heart rate, plasma OXT↑ Using OXT antagonist attenuates tachycardic responses	[101]
Rat, F	Psycho-social stress	OXT release in PVN↑	[171]
Human, F Mice, M	PTSD	Blood OXTR mRNA concentrations in human↑ Prefrontal cortical OXT and OXTR mRNA in mice↑ HPA axis response relies on regulation of OXTR expression	[157]
Human, M&F	PTSD	Plasma OXT in men and women ↓	[134]
Human, F	Acute stress (emotionally stressful video)	Negative emotion and salivary cortisol ↑ Baseline OXT predicts stress response	[172]

### Oxytocin and stress-related neuropsychiatric disorders

#### Anxiolytic role of oxytocin

Anxiety disorder is characterized by excessive fear, anxiety and avoidance of stress, including: separation anxiety disorder, selective mutism, specific phobias, social anxiety disorder (SAD), panic disorder, agoraphobia, and generalized anxiety disorder [102]. In the previous studies, OXT has been demonstrated to have anxiolytic effects on different types of anxiety. Separation anxiety disorder is the presence of inappropriate and excessive fear or anxiety while leaving the familiar environment or being separated from the attachment object. In lactating rats, long-term MS altered maternal care, anxiety-like behavior, and paraventricular OXT and corticotropin-releasing factor immunoreactivity expression in lactating rats [103]. At postnatal day 21, rat pups were experienced MS stress, resulting in anxiety behaviors with higher levels of blood CORT and impaired social and learning and memory functions [104]. Intranasal OXT administration rescued anxious behavior by restoring impaired LTP differentiation in hippocampal CA1 region [104]. Acute injection of fibroblast growth factor 2 (FGF2) was associated with the acH3K14 of the OXTR promoter in short term MS rat pups, suggesting that FGF2 mediates the anxiolytic effects by increasing acetylation of OXTR promoters to overcome decreased OXT levels [105]. In contrast, repeated daily experiences of brief separation from pups resulted in increases the level of estrogens receptor (ER) and OXTR and a decrease in 5-HT1Ars in the brain of rat dams [106]. Neurochemical changes may be responsible for the observed increase in maternal behavior and decreased anxiety [106]. However, MS after exposure to stressful environment during pregnancy will reduce stress susceptibility and anxiety behavior of offspring, and the mechanism may be related to changes of the serum concentration of 17-beta-oestradiol, OXT and Erβ/Erα ratio [107]. Further, our previous study designed that rat pups were subjected to early stress, and then exposed to restraint stress in adulthood to observe the stress sensitivity and susceptibility of rats. We found that predictable MS increased stress resilience in adulthood, while unpredictable MS increased susceptibility in “two-hit” stress model [9].

In clinical use, salivary OXT levels were inversely correlated with separation anxiety symptoms and be positively associated with the separation anxiety scores in children [108]. Thus, OXT has a potential implication for adolescent anxiety and treatment, especially those who have experienced childhood separation from their mothers [109]. Interestingly, OXTR and G protein β3 subunit genes were specifically associated with the presence and severity of MS, but this specificity is not associated with anxiety and depression [110].

SAD is characterized by a marked and persistent fear of social situations or potentially embarrassing social behaviors and activities, and features of avoidance behavior. Given its effects on social functioning and behavior, there are several studies about OXT use in SAD. An fMRI study of fearful face processing found decreased functional connectivity between the amygdala and anterior cingulate gyrus in the SAD patient compared to healthy controls with elevated anxiety symptoms [111]. However, intranasal OXT normalized the decreased connectivity between the amygdala and anterior cingulate gyrus in the SAD patient [111]. Plasma OXT levels were higher in SAD patients with more severe social anxiety symptoms, possibly because the high levels of oxytocin secretion in SAD patients compensated for reduced social anxiety symptoms [112]. In another study of patients with SAD, intranasal administration of OXT enhanced other-oriented reward motivation in patients with lower anxiety but higher anxiety, suggesting that OXT prosocial effects are related to the severity of symptoms [113]. Oxytocin may be a predictor of social anxiety disorder. Although the above-mentioned preclinical and clinical studies have provided the evidences of OXT in the intervention and biomarkers of different anxiety, the biological mechanism and neural activity of OXT have not been fully elucidated.

#### Oxytocin mediated improvement of depressive behavior

Depression is a stress-related neuropsychiatric disorders characterized by lower mood, thought retardation, loss of interest, and reduction of action and language, accompanied by suicidal attempts [114]. A previous study found that serum OXT was significantly negatively correlated with Hamilton Depression Scale score [115]. Another study of postpartum depression used the Edinburgh postnatal depression scale (EPDS) to assess the risk of postpartum depression and showed that plasma OXT plasma OXT concentrations were lower in the high-risk group compared with subjects in the low-risk group for postpartum depression [116]. The relationship between OXT and depressive behavior in animals was first reported in 1980s via i.c.v injection of OXT [117]. Then, Arletti et al. found that intraperitoneal injection (i.p) of OXT (0.25–1.0 mg/kg) could significantly improve depressive behavior [117]. And OXT was shown to like the pharmacological effects of impramine (7.5–30 mg/kg i.p) in depressive mice [117]. In the desperate behavior of rats, i.c.v injection of OXT reduced the immobility time of the forced swimming test and tail suspension test of rats via a dose-dependent effect [118]. The results shown that behavioral despair promote the synthesis and secretion of OXT in the brain and periphery [118]. But brain-derived OXT played a role in behavioral hopelessness depression, rather than the periphery-derived OXT [118]. In a socially isolated model of depression, OXT mediated the development of depressive-like behaviors following neuronal injury in mice [119]. The preventative pharmacotherapy of OXT can also attenuated the depressive behavior and deterioration of mood in male and female rats after early life stress [120]. I.c.v injection of oxytocin reversed depression-like behavior in a rat model of postpartum depression by regulating the HPA axis and TrkB in PVN signaling pathway [121, 122]. In clinical studies, it was found that OXT concentrations in the plasma of depressed patients were higher than those in healthy controls [123]. Intranasal oxytocin had no effect on facial emotion recognition and was associated with a reduction in negative thoughts in mothers with postpartum depression [124]. Interestingly, serum OXT levels during pregnancy were associated with depressive symptoms in early pregnancy or postpartum and may serve as a predictive target for postpartum depression [116, 125]. A allele of rs53576 in the oxytocin gene was also found to be associated with suicidal behavior in people with a history of suicide attempts, suggesting that OXT can also be used as a potential target for predicting suicide attempts [126]. However, there was no significant difference in the level of intravenous OXT in women with postpartum depression and healthy subjects measured through the Beck Depression Inventory II scale [127]. A meta-analysis also found that plasma OXT levels in depressed patients were not different from healthy subjects, suggesting that the effect of OXT needs to consider the heterogeneity of subtypes and samples of depressed patients [128].

Despite so much preclinical and clinical evidences for the intervention effects of OXT in depression, its biological mechanisms have not been systematically elucidated. The regulation of serotonergic function and HPA axis may mediate the antidepressant mechanism of OXT. Increased the expression of 5-hydroxytryptamine receptors and serotonin transporter of hippocampus correlated with oxytocin levels in a MS induced animal model of depression [129]. But OXT exerted a synergistic function of antidepressant efficacy, such as increasing SSRI sensitivity by regulating Integrin β3 (ITGB3) and Close homolog of L1 protein (CHL1) in BDNF expression [130]. Similarly, OXT can modulate stress behaviors and autonomic nervous system responses by attenuating HPA axis activity, thereby reducing depressive behaviors [88]. Infusion of oxytocin (100 ng/h, i.c.v) in ovariectomized rats reduced plasma ACTH concentrations and CRF mRNA in the hypothalamus after 30 min stress stimulation [88]. Also, MS-induced depression-like behaviors were attenuated by OXT mediated improvement of mitochondrial function and immune-inflammatory response in the hippocampus [35]. In terms of gene polymorphisms, some studies have found that the polymorphism of oxytocin-related genes (rs2254298) has a predictive effect on anxiety and depression symptoms in adolescent girls [131]. Catherine et al. found that the reduction of OXTR gene DNA methylation is related to perinatal stress and postnatal depression, while the increase of DNA methylation is related to social cognition and emotional recognition disorders [132]. Generally, there are differences in the evidence that OXT improves depression, but this may be related to the heterogeneity of the pathogenesis of depression per se. The mechanism of oxytocin improving depressive behavior also requires further exploration at the neuronal and molecular levels. Achieving the best therapeutic effect, as well as the intervention of different depression subtypes, needs more attention in future work.

#### The potential of oxytocin treatment in PTSD

After experiencing traumatic events, individuals are prone to the PTSD symptoms of social avoidance, cognitive and emotional changes, and hyperarousal. And it have been shown that the potential of OXT for the treatment of PTSD. In a meta-analysis, OXT was found to be effective in PTSD interventions [133]. Plasma OXT Levels of PTSD patient was lower than healthy subjects [134]. Intranasal OXT has been shown to improve social cognitive abilities such as emotion recognition, interpersonal trust, and prosocial behaviors, which are often impaired in patients with PTSD [135]. In the brain, OXT enhances functional connectivity between the amygdala and prefrontal regions and suppresses fear responses in the amygdala, which in turn is involved in the regulation of salience processing [136]. In male patients, OXT may reduce the control of CeM via enhancing mPFC regulation [72]. And in female patients, it reduces the control of amygdala in PTSD by reducing the projection of basolateral amygdala (BLA), which can attenuate anxiety and decreased fear expression [72]. The results implied that sex differences underly PTSD-related neurobiological mechanisms[72]. The patient with PTSD always exhibited social impairment and with the symptoms of emotional and cognitive empathy deficits [137]. Although intranasal OXT (24 IU) administration does not improve emotional cognitive empathy, it can selectively enhance the perception of angry body movements in man PTSD patients [137]. In Chinese earthquake survivors, serum OXT levels were not associated with overall PTSD symptoms and were associated with PTSD anxiety arousal symptoms [138]. But in the women patient with PTSD, the results indicated that a single intranasal dose of OXT enhances empathy for women [139]. OXT appears to affect males and females with PTSD differently, implying a need for gender-specific treatment in this population. In a study of OXT and Prolonged Exposure co-intervention in PTSD, it was found that intranasal OXT (40 IU) administration reduced PTSD and depressive symptoms in PE treatment, but there was no statistical difference [140]. A recent study also found that OXT is a potential biomarker for PTSD subtypes with high HPA response [66]. In the HPA axis subgroup, blood OXTR mRNA was associated with PTSD symptoms, and can predicted the activation levels in the prefrontal cortex of mice with PTSD-like symptoms [66].

In the rodent, moderate restraint stress increased social support-seeking behavior and decreasing the aggression by the activation of hypothalamic OXT signaling [141]. Conversely, after experiencing the stress contents of predator odor, the results have shown that social affiliation was disrupted (a PTSD-like symptom) with reduced OXT signaling in rat[141]. OXT administered immediately after trauma produced a short-term increase in recall of traumatic memories, whereas chronic administration of low-dose OXT had cumulative anxiolytic effects in social co-housing in a rodent model of PTSD [142]. In SPS, an animal model that effectively mimics PTSD, intranasal OXT administration reversed SPS-induced fear extinction repair and downregulated the levels of inflammatory factors (IL-1β, TNF-α) in the hippocampus and serum [63]. Meanwhile, OXT also promoted the recovery of SPS-induced social behavior abnormalities through the OXTR binding in mPFC and amygdala [64]. Interestingly, in the SPS model, OXT signaling also attenuated fear acquisition in the social-sexual partner pairing of prairie vole, providing a buffer for partner absence [143]. As mentioned above, OXT plays a potential role in social behavior via regulating stress response of PTSD. But given the heterogeneity of PSTD, the construction of animal models provides obstacles to the exploration of the biological mechanism in therapeutic uses of OXT.

### The augmentation of oxytocin expression

**Fig. 2:**
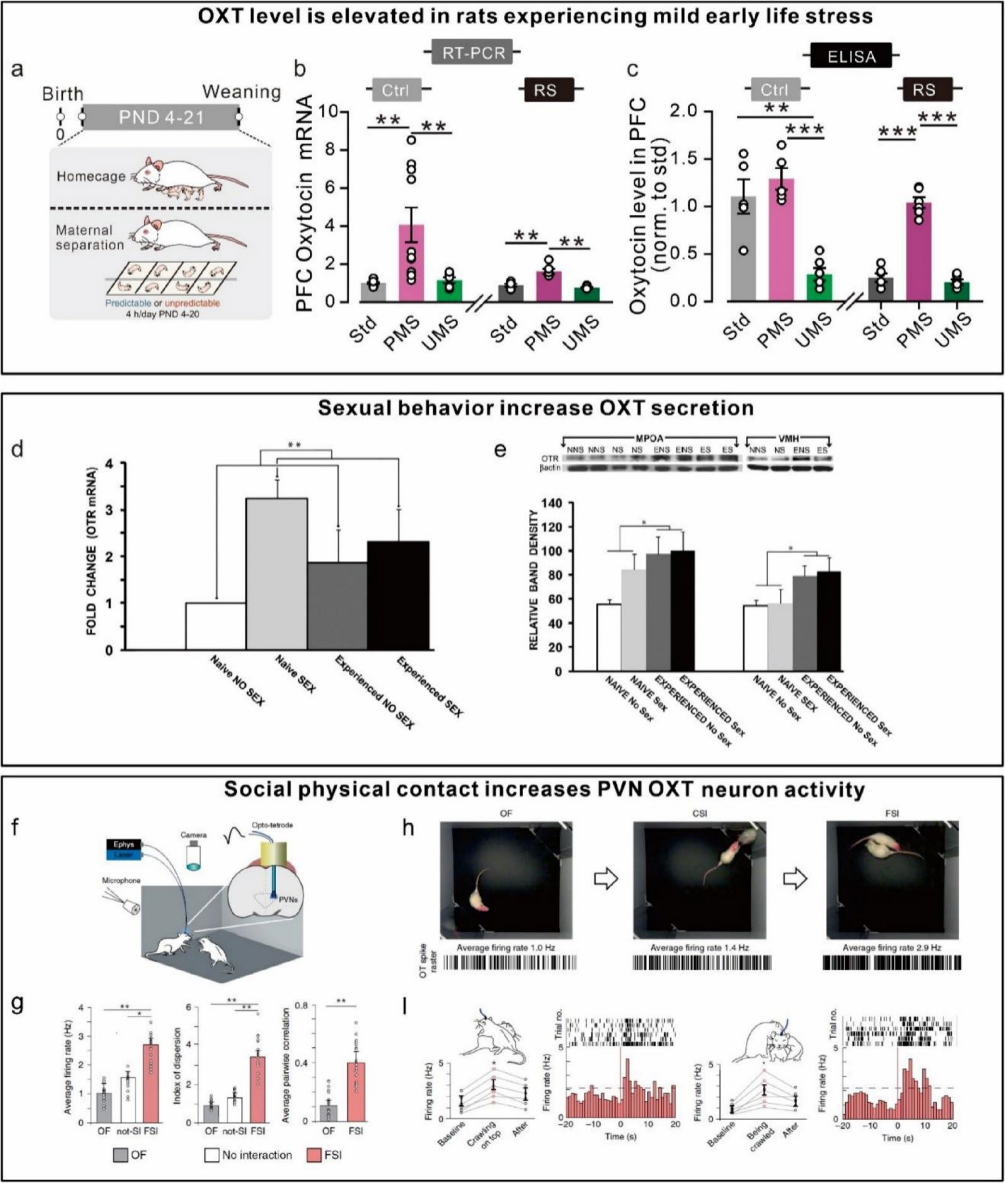
The augmentation of Oxytocin expression. **a** a illustration of early life stress. **b** and **c**, both oxytocin mRNA level and protein level were significantly increased in predictable maternal separation (mild early life stress), while were decreased in unpredictable maternal separation. a-c adapted from [9]. **d** copulation (i.e., acute effects of sexual activity) increases OXTR mRNA levels in the medial preoptic area (MPOA). **e** Previous sexual experience increases OXTR protein levels in the MPOA, ventromedial hypothalamus. **d** and **e** adapted from [148]. **f** Setup for recordings of behavior, ultrasonic vocalizations and neural activity. **g** Average firing rate of 15 OXT neurons from 5 rats. Free social interactions (FSI) led to the greatest change in firing rate and synchronous activity in simultaneously recorded OXT neurons. **h** Frames of recorded videos (top) of experimental rats that were placed either alone (Open field exploration), or with a mesh between rats (Chambered social interaction) or for FSI with a stimulus rat; representative spike raster plots of an OXT cell in each condition (bottom). experimental and stimulus rats were separated by a transparent wall with small holes (7.5 mm), allowing rats to see, sniff and hear, but not touch, each other. **i** Representative spike raster plots, averaged response and peristimulus time histograms of OXT cell activity during ‘crawling on top’. **f**–**i** adapted from [151]

The secretion of endogenous OXT may serve as a predictive target for stress-related neuropsychiatric disorders and may also provide buffers for stress-induced neuropsychiatric disorders. Inducing the release of OXT in the brain may produce a safe and effective anti-stress effect via stress response, maternal behavior, sexual behavior, social behavior and physical activity (Fig. 2).

Firstly, OXT levels are regulated by stress response. The obvious part shown that both chronic stress and acute stress can increase OXT expression in humans and animals, although this result is different in human studies, indicating that changes in OXT levels may be heterogeneous with disease. And OXT may be affected at different stages of the disease pathological process. Therefore, the expression of OXT should take into account these factors, such as the type of stress, the subtype of stress related neuropsychiatric disease, and the population differences. For instance, we found elevated levels and expression of OXT in the blood and mPFC of rats in a model of predictable MS, whereas the opposite effect was exhibited in unpredictable MS [9]. Stress-induced increase in OXT secretion was involved in increased cortisol responsiveness and recovery of vagus [144].

Secondly, pregnancy, lactation, and sexual behavior can increase OXT secretion in central and peripheral. OXT neurons were involved in the process of milk secretion in rats [145]. Inhibition of OXT neurons also reduced prolactin levels, suggesting that oxytocin neuron activation promotes prolactin activation during pregnancy and lactation [145, 146]. Interestingly, virgin mice, mother mice and pups were co-housed and the maternal behavior of the mother mice would be perceived by the virgin mice through visual learning [147]. Then maternal behavior promotes the virgin mice to react with their companion pups [147]. Through recordings of PVN neural activity, it was found that OXT neuron activation in PVN modulates increased plasticity in left auditory cortex and promotes alloparenting in virgin mice [147]. During sexual behavior, sexual experience increased the expression of oxytocin gene and protein in the medial preoptic area of male rats [148]. Conversely, intracerebral OXT injection also promoted sexual behavior in male rats, regardless of previous sexual experience [148]. And sexual self-stimulation also increases salivary OXT levels in healthy adult men and women [149]. Sexual behavior of male rats OXT release mode is not only synaptic axonal release but also occurs exocrine vesicle transmission and diffusion locally acts on OXTR neurons and promotes male sexual function [150].

Thirdly, Social touch induces activation of parvocellular OXT neurons in a small population of the hypothalamus and transmission to magnocellular neurons in rats [151]. Feedback regulation of social behavior by OXT neurons has also been validated in a mice model of autism [152, 153]. Lastly, some evidence suggested that exercise also modulates changes in OXT levels and OXT expression. In rats with gastric injury, regular, moderate-intensity exercise upregulates oxytocin activity and alleviates gastric injury [154]. Similar results were obtained in a mouse model of breast cancer [155]. Interval exercise training reduced the activity of PI3K/Akt and ERK through OXT secretion, thereby reducing tumor volume and weight [155]. Serum OXT levels in well-trained male and female runners rose from 1.5 pg/ml at rest to 3.5 pg/ml after prolonged, high-intensity endurance running [156]. In conclusion, OXT expression is affected by multiple factors, and increasing OXT levels and expression in a healthy, endogenous manner may improve resistance to stress-induced neuropsychiatric diseases.

## Concluding remarks

**Fig. 3:**
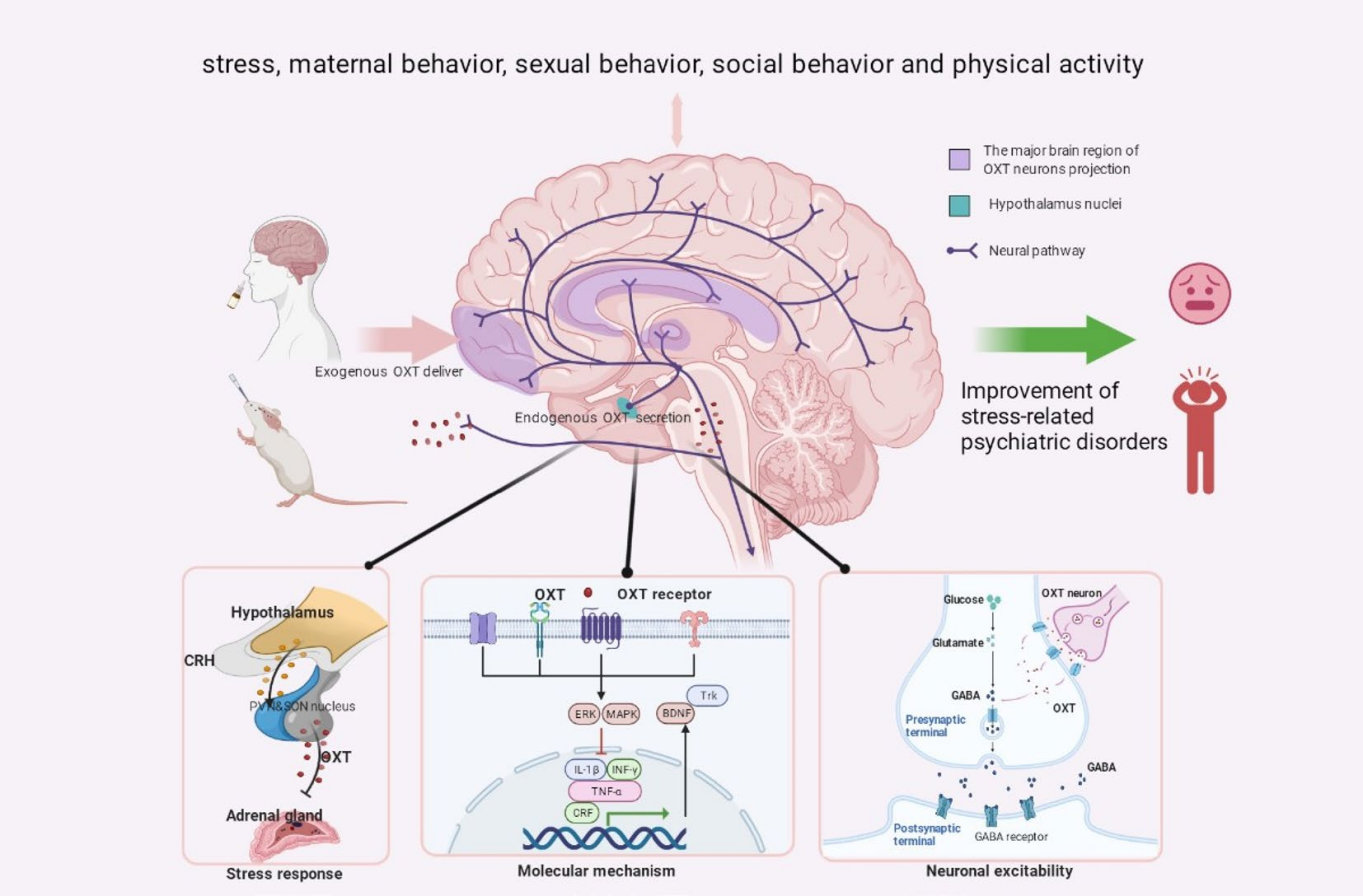
The regulation of endogenous and exogenous OXT in stress-related neuropsychiatric diseases and the bidirectional influencing factors of oxytocin expression in the brain. In humans and animals, intranasal OXT administration and other stimuli, such as stress, maternal behavior, and sexual behavior et al., induce elevated OXT levels that project primarily to the prefrontal cortex, hippocampus, and amygdala. The interaction of OXT with OXTR increases stress resistance and improves stress-related neuropsychiatric diseases by regulating stress response, signaling pathways, neuronal excitability. Image created with Biorender.com

Growing evidence has shown that OXT has an important role in stress-related neuropsychiatric diseases. To understand the effects of OXT in psychiatric disorders, this article reviews the OXT system and the main brain regions where OXT neurons project in stress. Based on recent evidence, we have also shown that targets and predictive roles of OXT signaling and neuronal interventions in stress-related neuropsychiatric disorders. Finally, we propose several methods to increase the expression of OXT to provide ideas for follow-up research (Fig. 3).

Although there are many evidences for OXT and stress-related neuropsychiatric disorders, there are still many puzzles waiting for us to solve. As a potential target of neuropsychiatric diseases, how OXT signaling plays its role in improving neuropsychiatric diseases needs to be further explored and fully grasped in future research. Given the complexity and heterogeneity of stress-induced neuropsychiatric disease processes, the following questions need to be addressed. Firstly, whether the molecular pathways mediated by peripheral OXT levels differ from those in central oxytocinergic neurons. Secondly, the dose-dependent effect of OXT needs to be further clarified in different diseases, populations, and animal models. Thirdly, how OXT neuronal activity and molecular changes are linked to stress-induced neuropsychiatric disorders. The elaboration of the above questions can make better use of OXT as a target to further improve neuropsychiatric diseases and provide stronger evidence for the mechanism.

## Data Availability

The authors declare the availability of data and material.

## Abbreviations

BNST: Bed nucleus of the stria terminalis
CeA: Central amygdala
CORT: Corticosterone
CREB: CAMP response element-binding
CRH: Corticotrophin-releasing hormone
CRHBP: Corticotropin releasing hormone binding protein
CRHR: CRH receptors
CRTC: CREB-regulated transcriptional coactivators
DAG: 1,2-Dicyaglycerol
DG: Dentate gyrus
EGFR: Epidermal growth factor receptor
EPDS: Edinburgh postnatal depression scale
ER: Estrogens receptor
ERβ: Estrogen receptor beta
FGF2: Fibroblast growth factor 2
GPCR: G protein-coupled receptor
i.c.v injection: Intracerebroventricular injection
i.p injection: Intraperitoneal injection
IP3: Inositol 3 phosphate
MAPK: Mitogen-activated protein kinase
MS: Maternal separation
OXT: Oxytocin
OXTR: Oxytocin receptor
OXTRINS: OXTR interneurons
PD: Paternal deprivation
PLC: Phospholipase C
PTSD: Post-traumatic stress disorder
PVN: Paraventricular nucleus
SAD: Social anxiety disorder
SON: Supraoptic nucleus
SPS: Single prolonged stress
TLR4: Toll like receptor 4
TRPV2: Transient receptor potential vanilloid type 2
vlPFC: Ventromedial prefrontal cortex
